# Impact of Pretreatment and Drying Factors on Chemical and Biochemical Attributes of Moroccan *Thompson Seedless* Grapes

**DOI:** 10.1155/2023/4438353

**Published:** 2023-11-15

**Authors:** Abdelhakim Boudboud, Mohamed Ben Aziz, Hassan Hajjaj, Lhoussain Hajji, Bruno de Meulenaer, Hamid Mazouz

**Affiliations:** ^1^Moulay Ismail University, Faculty of Sciences, Laboratory of Biotechnology and Bioresources Valorization, BP 11201, Zitoune, Meknes, Morocco; ^2^Moulay Ismail University, Cluster of Competency “Agri-food, Safety and Security”, Marjane 2, BP 298 Meknes, Morocco; ^3^Sultan Moulay Slimane University, High School of Technology, Laboratory of Biotechnology, Bioresources and Bioinformatics, Khenifra, Morocco; ^4^Ghent University, Faculty of Bioscience Engineering, Department of Food technology, Safety and Health, Research Group Food Chemistry and Human Nutrition (NutriFOODchem), Ghent, Belgium

## Abstract

Drying is a common technique in the agrifood industry, but insufficient control in the drying process can result in changes to the fruit's appearance due to physiological damage during processing. The aim of this study was to investigate the impact of pretreatment and drying process parameters on Moroccan raisins' quality and safety. The experimental levels of pretreatment factors (blanching, browning agents) and drying temperature were defined at the beginning. Subsequently, a 2^4^-factorial design was employed to provide a simple and reliable model capable of relating directly the response factor (drying time, color intensity change (*E*^∗^), chromaticity (*C*^∗^), and browning rate) to the variables (NaOH concentration, antibrowning agent concentration, temperature, and relative humidity). All four parameters had a statistically considerable effect on studied responses. Blanching for 5 minutes at 1% of NaOH solution, using an appropriate concentration of antibrowning agent (5% Na_2_S_2_O_5_), and drying at 70°C with 30% of relative moisture can lead to better preservation of grapes' appearance and quality (chromaticity (*C*^∗^) and color change (*E*^∗^)). Also, in these conditions, a lower browning rate (14.48%), a lower 5-hydroxymethylfurfural content (12.40 mg/100 g DW), and a higher level of polyphenols (135.79 ± 13.17 mg GAE/100 g DW) and flavonoid content (57.81 ± 3.08 mg Qeq/100 g DW) have been recorded while meeting international standards for SO_2_ content and microbial quality.

## 1. Introduction

Grapes hold a prominent position among globally cultivated fruits, with a staggering 84.7 million tons produced worldwide in 2021 [1]. Morocco, while ranking 27^th^ in global grape production with 420,113 tons in 2021 [1], concentrates its grape cultivation predominantly in the central region, encompassing Doukkala, Haouz, Benslimane, and Rabat-Sale [2]. *Thompson Seedless* grapes, celebrated for their nutritional qualities and versatility in various food applications such as yogurt, ice cream, and cakes [2–4], are especially esteemed in Morocco. These grapes are replete with health-enhancing bioactive compounds, including polyphenols, carotenoids, and vitamins [5]. Yet, the high sugar and moisture content of these grapes renders them susceptible to microbial degradation, necessitating rapid consumption or processing to curtail economic losses [6]. To this end, drying emerges as the prevailing method to convert grapes into raisins, effectively extending their shelf life across production regions [7–9]. The fundamental objectives of this process are to reduce water content and water activity (*a*_w_) to safe levels, thereby enabling the storage of dried grapes at room temperature over an extended duration [6, 8]. Intriguingly, although Morocco excels as a producer of fresh *Thompson Seedless* grapes, the majority of golden raisins are entirely imported. Imports have surged consistently over the years, soaring from 563 tons in 2016 to a staggering 12,418 tons in 2021 [10], underscoring their popularity among Moroccans. This surging demand presents an opportunity for local production, leveraging Morocco's abundant resources. However, a deficiency in drying expertise poses a formidable obstacle. Inadequate process control can lead to the deterioration of grape compounds due to prolonged drying times and oxidation, resulting in alterations to color, texture, taste, and nutritional value of raisins [11]. These transformations, notably surface browning, limit consumer acceptance. This is why a profound understanding of operational parameters of drying [12], which is tightly linked to operational treatment parameters like blanching, drying temperature, and relative humidity, is crucial [7]. A central challenge in this context revolves around the intrinsic attributes of grapes, including volume, berry size, sugar concentration, maturity, and notably, the presence of a wax outer peel layer [11, 13]. This wax layer acts as a barrier to moisture movement across the grape's membrane, intensifying the complexity of the drying process. Consequently, a plethora of pretreatment techniques has been extensively explored in scientific literature to expedite grape drying and raisin production. Notably, Wang et al. [14] investigated the efficacy of high-humidity hot air impingement, seeking to enhance the drying process. Chemical pretreatments, such as grape immersion in an alkaline emulsion containing ethyl oleate, K_2_CO_3_ [9], and NaOH, have garnered significant attention. These chemical pretreatments primarily are aimed at eliminating the waxy layer on grape skins while concurrently mitigating microbial activity during subsequent drying stages [15, 16]. The application of NaOH treatments, in particular, is a prevalent practice in large-scale raisin production. This method is renowned for its capacity to amplify drying efficiency while simultaneously enhancing the color of dried grapes, as demonstrated by Vázquez et al. [16]. This beneficial effect is likely attributed to the softening of the grape skin, which facilitates permeability, subsequently expediting the drying process, as proposed by Zemni et al. [17]. Following blanching, the subsequent step involves immersing grapes in an antibrowning agent to preserve their color by deactivating PPO and POD enzymes responsible for color alteration [18]. The study is aimed at exploring how pretreatment and drying conditions affect the chemical and biochemical properties of Moroccan *Thompson Seedless* grapes, using a 2^4^-factorial design to analyze the effect of factors like NaOH concentration, antibrowning agent concentration, temperature, and relative humidity on drying time, color change (*E*^∗^), chromaticity (*C*^∗^), and browning rate. The final step entails evaluating the quality of dried grapes under optimal conditions, ensuring compliance with international standards for dried grapes.

## 2. Materials and Methods

### 2.1. Chemicals

All chemicals' products used in this work were provided by Sigma-Aldrich, except otherwise stated in the text.

### 2.2. Raw Material Origin and Sampling

In order to select grapes with high-level potential for dehydration, grapes (*Vitis vinifera Thompson Seedless*) were collected at commercial maturity from three Morocco localities: Meknes, El Hajeb, and Nador cities. The largest nearby market was the source of three sets of fruit samples that were taken during specific time intervals. The fruits chosen for sampling were scrutinized for their firmness, color, and any visible blemishes before being placed in polyethylene bags. Subsequently, the fruit samples were kept in cold storage at 4°C until the time for processing or analysis arrived.

### 2.3. Residual Activity after Inhibition Treatments of PPO and POD Enzymes

In order to select optimum blanching duration, grapes were soaked in solution of 0.5 and 1% of NaOH at 90°C between 1 and 10 minutes. The enzymes were extracted from the blanched grapes in the McIlvaine buffer according to the method of Deng et al. [19], then, the activity of PPO and POD were measured based on the methods described by Gonçalves et al. [20] and Wang et al. [21]. The inhibition rate was calculated according to the following equation (the same equation was used to determine the inhibition rate of PPO after drying in optimum conditions):

(1) $$\begin{array}{c} \%\text{of residual activity}=100-\left(\frac{\left(\text{AUi}-\text{AUf}\right)}{\text{AUi}}*100\right), \end{array}$$

where AUi is the initial enzymatic activity without treatment and AUf is the enzymatic activity of blanched grapes or dried grapes.

### 2.4. Drying Process of Grapes

**Figure 1:**
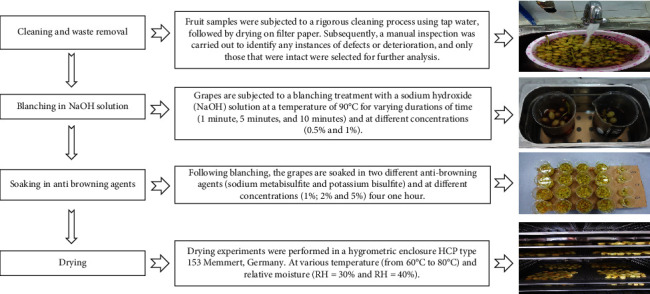
Description of the experimental procedure used for the drying of grapes (*vr. Thompson Seedless*).

As mentioned in Figure 1, the fruit samples (approximately 1 kg in each test) were subjected to a rigorous cleaning process using tap water, followed by drying on filter paper. Subsequently, a manual inspection was carried out to identify any instances of defects or deterioration. After undergoing the cleaning process, grapes are subjected to a blanching treatment with a sodium hydroxide (NaOH) solution at a temperature of 90°C, for varying durations of time (1 minute, 2 minutes, and 10 minutes) and at different concentrations (0.5% and 1%). Following by blanching, the grapes are soaked in two different antibrowning agents (sodium metabisulfite and potassium bisulfite) for one hour and at different concentrations (1%, 2%, and 5%). After the soaking process, the samples are decanted. Drying experiments were performed in a Hygrometric enclosure HCP type 153 (Memmert, Germany), at various temperature (from 60°C to 80°C) and relative moisture (RH = 30%andRH = 40%), until reaching a moisture content value of 18%, which is the maximum moisture content required by standard for dried grapes [22].

### 2.5. Experimental Design to Determine Optimum Conditions for Drying Grapes

In order to determine optimal conditions for drying grapes, a 2^4^-factorial design was employed to analyze the effects of selected variables: NaOH concentration (*X*_1_), antibrowning agent concentration (*X*_2_), temperature (*X*_3_), and relative humidity (*X*_4_), with their respective levels presented in Table 1, on the observed responses (Table 2), which included drying time (*Y*_1_), color intensity change *E*^∗^ (*Y*_2_), chromaticity *C*^∗^ (*Y*_3_), and browning rate (*Y*_4_). The experiments were performed according to the matrix presented in Table 2. The final step involved evaluating the quality of the dried grapes under optimal conditions.

### 2.6. Physicochemical Analysis

#### 2.6.1. Chromatic Characteristics of Fresh and Dried Grapes (*vr. Thompson Seedless*)

The surface color of dried apricot samples was measured by a spectrophotometer Minolta CR:5 to determine the lightness (*L*^∗^), redness (*a*^∗^), and yellowness (*b*^∗^). Saturation (chroma, *C*^∗^), total color change (*E*^∗^), and browning rate were generated by *L*^∗^, *a*^∗^, and *b*^∗^ color coordinates using the following equations [23]:

(2) $$\begin{array}{c} C*=\sqrt[{2}]{\left(a^{*2}\right)+\left(b^{*2}\right)},\\ E=\sqrt[{2}]{\left({\Delta L}^{*2}\right)+\left({\Delta a}^{*2}\right)+\left({\Delta b}^{*2}\right)},\\ \text{Browning rate}=\frac{L*-L}{L*}\times 100,\\ \text{Yellow index}=\frac{142,86*b}{L.}. \end{array}$$

#### 2.6.2. Water Content

Water content was measured with a moisture determination balance (Vibra, Md series); briefly, the samples are put into the machine in a small stainless steel tray, the weight of which is already taken by the machine; the sample is heated in the chamber to a temperature of 100°C, and each time the weight is measured until it has a stable value.

#### 2.6.3. Water Activity

The *a*_w_ is determined at 25°C with an *a*_w_ meter (AquaLab CX-2, USA).

#### 2.6.4. Soluble Solids

Grape samples are homogenized and filtered; then, °Brix was estimated using a refractometer (ATAGO Pocket Digital) at 25°C.

#### 2.6.5. pH

A quantity of 20 g of grape samples was homogenized for five minutes and filtered using a paper filter. The filtrate was analyzed to determine its pH value using a pH meter from Weilheim, Germany.

#### 2.6.6. Rehydration Capacity (RC)

To rehydrate the samples, they were immersed in distilled water at a temperature of 25°C for five hours. The grape-to-water ratio used was approximately 1 : 30, according to the methodology employed by [24]. The RC was expressed as the percentage of water gain and was measured using the following equation:

(3) $$\begin{array}{c} \text{RC}\left(\%\right)=\left(\frac{\text{WR}-\text{WS}}{\text{WS}}\right)*100, \end{array}$$

where WR is the rehydrated grape weight and WS is the samples before rehydrated weight.

### 2.7. Biochemical Characteristics of Fresh and Dried Grapes (*vr. Thompson Seedless*)

#### 2.7.1. Total Phenol Content (TPC)

Polyphenols were extracted according to the method described by [25]. 2 g of samples was homogenized with methanol (80%) for 30 min and centrifuged at 4500 tr/min. The supernatant was used to quantify a total phenolic compound according to the Folin–Ciocalteu method [26]. 250 *μ*l of extract was mixed with 250 *μ*l of the Folin–Ciocalteu reagent and 500 *μ*l of Na_2_CO_3_ (20%). The sample was thoroughly mixed and incubated in a water bath for 30 min at 37°C. Subsequently, the absorbance at 750 nm was measured by a spectrophotometer (V-630 UV-Vis Spectrophotometer JASCO (USA)). The result was expressed as milligram of gallic acid equivalent per 100 g of dry weight (GAE)/g DW.

#### 2.7.2. Total Flavonoid Content (TFC)

TFC were determined using the method explained by [27]; 500 *μ*l of the extract is added to 1500 *μ*l of methanol (96%), 100 *μ*l of 10% aluminum chloride (AlCl_3_), 100 *μ*l of sodium acetate (1 M), and 2800 *μ*l of distilled water. The mixture is stirred and incubated at room temperature in the dark for 30 min. The absorbance is measured at 415 nm using a spectrophotometer (V-630 UV-Vis Spectrophotometer by JASCO (USA)). The TFC are expressed in milligram of quercetin equivalent/100 g of dry weight, referring to calibration curve of quercetin.

#### 2.7.3. Pigment Analysis (chlr a-chlr b-chlr tot)

The process outlined by De-Kok and Graham [28] involved homogenizing five grams of grapes with 20 ml of 80% acetone using an Ultra-Turrax for five minutes. After centrifugation at 7,000 g and 4°C for 10 minutes, the supernatants were measured for absorbance at 663 nm and 645 nm to determine the levels of pigments. To calculate the parameters, standard equations from [29] were used.

(4) $$\begin{array}{c} \text{Total chlorophyll}=20.2\left(A_{645}\right)+8.02\left(A_{663}\right),\\ \text{Chlorophyll a}=12.72\left(A_{663}\right)-2.69\left(A_{645}\right),\\ \text{Chlorophyll b}=22.9\left(A_{645}\right)-4.68\left(A_{663}\right). \end{array}$$

#### 2.7.4. Determination of 5-Hydroxymethylfurfural (HMF)

The 5-hydroxymethylfurfural (HMF) analysis was achieved by using high-performance liquid chromatography (HPLC). Briefly, in the flask (50 ml), 5 ml of 0.3 N oxalic acid was added to 5 g of grape sample. The mixture was incubated for 60 min in boiling water bath. After incubation, 5 ml of 40% trichloroacetic acid was added and completed with distilled water until the mark, then filtered through a 0.45 *μ*m HPLC filter, and then injected in HPLC ultimate 3000 equipped with a column Lichrosorb RP-18 (250 × 4.6 mm, 5 *μ*m). A methanol water solution (90 : 10, *v* : *v*) was employed at a flow rate of 1.0 ml/min, and the detection was done at 285 nm.

#### 2.7.5. Residual Sulfur Dioxide in Dried Samples

The sulfur dioxide (SO_2_) content of the dried grapes was measured according to Monnier-William's distillation method [30, 31]. The results were expressed as milligram of SO_2_ per kilogram of dried grapes.

### 2.8. Microbial Quality Assessment of Dried Grapes under Optimal Conditions

The microbiological characterization of dried grapes stored under optimum conditions involved a one-year storage period at room temperature in airtight plastic bags. Two 10 g portions of each sample were placed into a sterile stomacher bag and processed according to the following protocol. One portion was diluted with buffered peptone water (1 : 9 *w*/*v* ratio) and homogenized with a stomacher for 60 seconds at 230 rpm. The resulting samples were processed for the enumeration of aerobic colonies [32], Enterobacteriaceae [33], Clostridium spp. [34], Bacillus spp. [35], coliforms [36], Escherichia coli [37], lactic acid bacteria [38], and the detection of Salmonella spp. [39]. The second portion was diluted with the Fraser Broth Base (1 : 9 *w*/*v* ratio) and homogenized with a stomacher at 230 rpm for 60 seconds. These samples were processed for the detection of Listeria monocytogenes [40]. The ISO standards were followed for all bacteriological determinations.

#### 2.8.1. Identification of Strains

To confirm the identification of a representative number of colonies from each medium, matrix-assisted laser desorption/ionization-time of flight mass spectrometry was employed according to Trabelsi et al. [41]. A portion of each colony or mycelium was placed on a VMS target slide and lysed with 1 *μ*l of 25% formic acid from bioMérieux. The lysate was then dried at 25°C, and 1 *μ*l of *α*-cyano-4-hydroxycinnamic acid matrix from bioMérieux was applied to each spot. The target slide, containing all the spots, was introduced into the VMS machine, which operated in positive linear mode with laser frequency of 50 Hz, acceleration voltage of 20 kV, and extraction delay time of 200 ns. The mass spectrum range was set to detect from 2000 to 20,000 Da. The generated mass spectra were unique for each spot and were compared to the reference spectra and super spectra database in the SARAMIS software, to obtain identification at the genus and species levels (a match more than 70% was deemed reliable).

### 2.9. Statistical Analysis

Grapes from each sample were homogenized, and then, three subsamples were selected for each of the three assays that were done. The ANOVA test was conducted by SPSS software, and results were shown as mean value ± standard deviation. The JMP 17 program was used to carry out the statistical analysis for the experimental design. A first-degree polynomial model was fitted to the independent and dependent variables. The impact of the variables on the various answers was then examined using the regression coefficients.

## 3. Results and Discussion

### 3.1. Biochemical Characterization of the Collected Fresh Grapes (*vr. Thompson Seedless*)

For the purpose of selecting grapes with optimal dehydration potential, three samples of *Thompson Seedless* variety from distinct regions and their biochemical characteristics and dehydration potential were evaluated (Table 3). Statistical analysis revealed that the factor “region” significantly (*p* < 0.05) impacted total soluble solids, chroma *C*^∗^, chlorophyll a and chlorophyll b, phenolic compounds, and PPO activity (Table 3). The water content of the samples ranged from 79.47 ± 1.69% (Nador region) to 81.77 ± 0.67% (Meknes region), which is within the range reported by Teixeira et al. [42] for 18 different grape cultivars (75.7-86.2%). The soluble solid content (Brix) is an important quality factor that significantly influences the color, texture, and overall sensory quality of the fruit [43, 44]. Brix levels were found to vary significantly among grapes from different regions, ranging from 16.37 ± 1.02% in the El Hajeb region to 25.33 ± 0.57% in the Meknes region. Grapes from the Meknes region recorded the lowest chlorophyll content (603.76 ± 11.37 mg/100 g FW) and the highest of polyphenol content (170.61 ± 0.62 mg GAE/100 g FW) and flavonoids (35.30 ± 0.34 mg Qeq/100 g FW). Decrease in the content of chlorophyll during the ripening of the grapes leads to an increase in the levels of carotenoids and anthocyanin pigments responsible for the change in their colors from green to yellow [45]. For the chromatic characteristic, it was observed that the grapes from Meknes region show a slightly higher chromaticity (21.47 ± 0.15) and highest yellow index (71.30 ± 1.61). This index is generally correlated with the pigment's contents responsible of Thompson Seedless color. Polyphenol oxidase (PPO) and phenol peroxidase (POD) are the focus of numerous studies because of their impact in food modifications and preservation. The PPO activity ranged from 14.59 ± 0.79 UIA/g.min to 18.95 ± 1.70 UIA/g.min in Nador region grapes and Meknes grapes, respectively. Grapes from the Meknes region possessed the highest levels of PPO activity (18.95 ± 1.70 UIA/g.min), and the difference of enzymatic activity observed between the three regions can be explained by the nature of these enzymes and the concentration of the substrates (phenolic compounds). Based on the better coloration, richness of phenolic compounds, and soluble solid content (Brix) in grapes from Meknes, it was chosen as the candidate for the remainder of this study.

### 3.2. Effect of Blanching Time on PPO and POD Residual Activities

**Figure 2:**
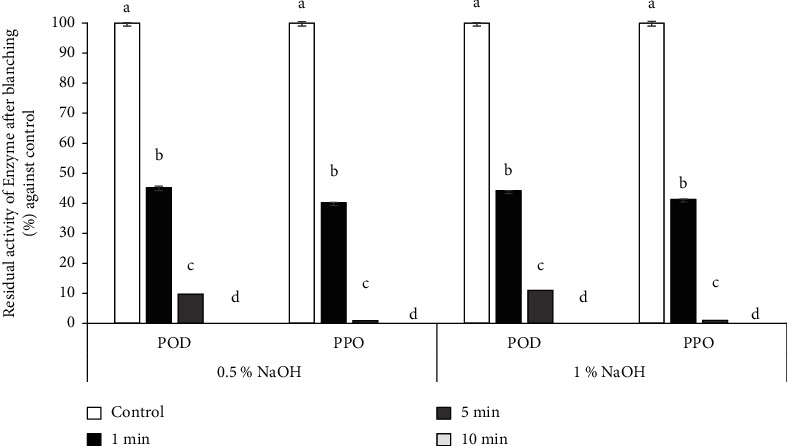
The residual activity of polyphenol oxidase (PPO) and phenol peroxidase (POD) after a blanching in a solution of NaOH (0.5% and 1% of concentration) for different duration.

PPO and POD are enzymes that cause browning and loss of color during drying grapes. PPO catalyze the oxidation of phenolic compounds in the presence of oxygen. POD enzyme catalyzes the oxidation of various substrates using hydrogen peroxide as a cosubstrate [46]. Blanching is one of the effective ways to control these phenomena [47]. In this study, the grapes were subjected to blanching treatments by immersing them in NaOH solution (0.5% and 1%) at 90°C for varying durations (1, 5, and 10 minutes). The impact of blanching treatments on PPO and POD enzymes' inhibition rate is investigated in Figure 2. The results indicate that the blanching treatments significantly reduce the activity of PPO and POD enzymes. After 5 minutes of soaking the grapes in NaOH at 90°C, the residual activities recorded of the two enzymes are less than 11% for POD and 2% for PPO in the two concentrations 0.5% and 1%, respectively (Figure 2). Blanching for 10 minutes effectively inactivated enzymes. But the grapes lose their texture at this duration. Similar findings were reported by Noreña and Rigon [48] and Mai et al. [49] regarding the effect of blanching duration on PPO and POD inhibition. During blanching, the heat denatures the PPO and POD protein structures and deactivates the enzymes by disrupting the weak bonds that hold the enzyme molecules together, resulting in the loss of their catalytic activity [50]. According to the result of Figure 2, blanching for 5 minutes in NaOH significantly reduces the enzymatic reactions at the same time preserves the structural integrity of the grapes. 5 min blanching duration was selected as a constant value for further experiments.

### 3.3. Selection of Drying Temperature and Antibrowning Agent Levels for Experimental Design

**Figure 3:**
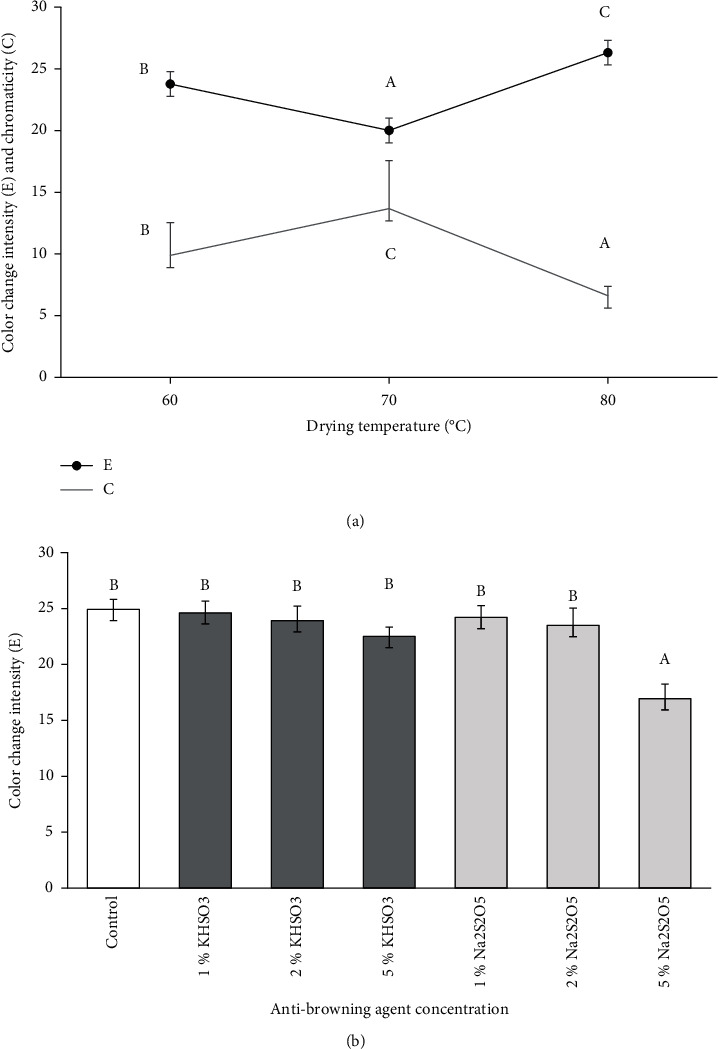
Effect of temperature (a) and antibrowning agent (b) on color characteristic of dried grapes (*vr. Thompson Seedless*).

In order to select the drying temperature and the best antibrowning agent between (Na_2_S_2_O_5_ and KHSO_3_), drying tests were done at various drying temperatures with different concentrations (Figures 3(a) and 3(b)). The results show a significant difference (*p* < 0.05) in the HunterLab parameters (*L*^∗^, *a*^∗^, and *b*^∗^) between fresh and dried grapes across all combinations tested. And according to the analysis of variance, the factors drying temperature had a higher impact on color intensity change and chromaticity *C*^∗^ (Figure 3(a)). As illustrated in Figure 3(a), the lowest chromaticity *C*^∗^ and highest intensity of color change *E*^∗^ which indicated a higher level of color degradation were observed at higher temperature (80°C). And according to Figure 3(b), the use of Na_2_S_2_O_5_ was more efficient in all concentration than the KHSO_3_. Furthermore, 2% of Na_2_S_2_O_5_ was the lowest concentration that preserve a characteristic color of *Thompson Seedless* grapes. Based on the results in Figure 3, the levels of drying temperature (60 and 70°C) and the Na_2_S_2_O_5_ concentration (2% and 5%) were selected to establish a factorial experimental design, in order to study their effects on the quality of our dried samples.

### 3.4. Experimental Design for Drying Grapes of *Thompson Seedless* Variety

The experimental design and the obtained results for drying treatments are presented in Table 4. Equations (5) to (8) show the obtained first-degree polynomial models for drying time (*Y*_1_), color intensity changes *E*^∗^ (*Y*_2_), chromaticity *C*^∗^ (*Y*_3_), and browning (*Y*_4_) responses, respectively, in relation to the significant variables and to the interaction between them at *p* < 0.05. Values of *R*^2^ represent the fit between the experimental data and the proposed model.

(5) $$\begin{array}{c} Y_{1}=33.5–7X_{3}+2.5X_{4}\;\left(R^{2}=0.99\right). \end{array}$$

Drying time (*Y*_1_) is crucial for the quality and safety of raisins, as it prevents spoilage and ensures safe consumption. Insufficient drying time can lead to bacterial growth, while excessive time can result in loss of flavor and nutrition. Meticulous monitoring of drying time is necessary to maintain appropriate moisture levels to meet the international standards of dried fruits [51]. Numerical coefficients in equation (5) show that the increase in temperature values (*X*_3_) and decrease in relative humidity (*X*_4_) have a positive effect on the drying time of grapes (*Thompson Seedless*). The minimum drying duration required to reduce the moisture content to 18% [51] was 23 hours achieved at a temperature of 70°C (Table 2). Generally, this result of drying time was slightly higher to the values cited by Doymaz and Mehmet [52] whom reported 20.5 hours for drying at 70°C. Esmaiili et al. [53] claimed 10 hours for drying times at 70°C. The difference between this result and ours can be explained by the variations between the physical properties of the grape and the technical aspects of the dryer employed in each of the studies.

(6) $$\begin{array}{c} Y_{2}=22.55–2X_{2}-1.1X_{3}-0.84X_{2}X_{3}+1.26X_{3}X_{4}\;\left(R^{2}=0.94\right), \end{array}$$

(7) $$\begin{array}{c} Y_{3}=11.73+2.02X_{2}+1.05X_{3}\;\left(R^{2}=0.95\right). \end{array}$$

Equation (6) presents the intensity of color change (*E*^∗^), which refers to the strength or magnitude of color change observed between two different color states. And Equation (7) described the chromaticity change which is the quality of the color regardless of its brightness or intensity; a dried grape with high chromaticity *C*^∗^ can be very bright and saturated. Equations (6) and (7) show that an increase in Na_2_S_2_O_5_ concentration (*X*_2_) and drying temperature (*X*_3_) result in less intensity of color change (*E*^∗^) and better preservation of chromaticity *C*^∗^, respectively. Increasing the concentration of antibrowning agents (Na_2_S_2_O_5_) reduced grape color degradation (decrease of chromaticity *C*^∗^).

(8) $$\begin{array}{c} Y_{4}=37.46-3.84X_{2}-2.46X_{3}+4.05X_{3}X_{4}\;\left(R^{2}=0.92\right). \end{array}$$

Equation (8) presents the browning index (*Y*_4_), which is a quantitative measure of the color alteration of dried fruits, used to evaluate their quality [54]. This measure is based on the quantification of browning reactions that occur during drying (enzymatic and nonenzymatic browning), which can significantly affect the appearance, texture, flavor, and nutritional value of dried samples [54]. Equation (8) shows that the increase in Na_2_S_2_O_5_ concentration and drying temperature (*X*_3_) and decrease in relative humidity (*X*_4_) result in less surface browning of dried grapes. Color preservation and reducing browning surface of raisins need an increase of Na_2_S_2_O_5_ concentration (*X*_2_) and drying temperature (*X*_3_) and a decrease in relative humidity (*X*_4_) (equations (6), (7), and (8)). The increase in temperature and the decrease in relative humidity can reduce the drying time (*Y*_1_) and the contact time of the grapes with the oxygen in the air, which is an important element for enzymatic browning reactions. Similarly, [48] observed that higher drying temperatures help to preserve color against degradation. Nearly the same temperature (75°C) was found by Doymaz and Altıner [55] to be the optimal drying temperature to preserve the color of raisin. The antibrowning agent (sodium metabisulfite (Na_2_S_2_O_5_)) is often used to release sulfur dioxide (SO_2_) [56], commonly used in the food industry to inhibit enzymatic browning and preserve color of fruit. In the context of grapes (*vr. Thompson Seedless*), the use of these agents can help to maintain the natural golden color during the drying process [16]. Sodium metabisulfite (Na_2_S_2_O_5_) reacts with water (H_2_O) (during soaking step) to form sodium hydrogen sulfite (NaHSO_3_) in aqueous solution and sulfur dioxide (SO_2_) in gaseous form. The higher Na_2_S_2_O_5_ concentration (5%) can preserve grape color from degradation. These results can be explained by the fact that a higher concentration of Na_2_S_2_O_5_ solution preserves more golden color through direct enzymatic inactivation of PPO, which occurs due to a modification of the tertiary structure of the PPO enzymes caused by the reduction of disulphide bond [57]. Furthermore, it is used also to control nonenzymatic browning, by its ability to act on different pathways and stages of this set of reactions (Maillard's reaction). It can react with carbonyl compounds to form hydroxy-sulfonates, blocking the appearance of brown products [58].

### 3.5. Physicochemical and Biochemical Parameters of Dried Grape Samples Dried under Optimal Conditions

Despite the technological effectiveness of sulphites on the preservation of the dried fruit quality, their use is regulated by the organisms such as Codex Alimentarius standards [59]. In order to ensure that our samples meet the international standards for raisins' qualities and food safety, the raisins treated according to the optimal parameters established by experimental design (concentration of NaOH (1%), blanching time (5 min), drying temperature (70°C), RH (30%), and 5% of Na_2_S_2_O_5_) are compared with grapes treated under the same conditions without the use of the antibrowning agent (0%) (control) (Table 5). The result shows that concentration of residual SO_2_ in treated samples was 580 ± 23.0 mg of SO_2_/kg. This value was lower 4 times than the maximum residual SO_2_ contents allowed by the Codex Alimentarius (2000 mg of SO_2_/kg) [59]. Water activity (*a*_w_) and water content are important parameters to consider grapes as they affect the quality, shelf life, and safety of the product [60]. The results showed that the moisture contents and *a*_w_ values of dried grapes were 17.90 ± 1.80% and 0.52 ± 0.06, respectively. Those results are in accordance with the Codex standard for dried grapes [51] which specified a maximum moisture content of 18% for seedless grapes. The use of the antibrowning agent increases the efficiency of the drying process; the grapes treated with 5% Na_2_S_2_O_5_ concentration dry faster than the untreated grapes (Table 5). Previous studies demonstrated a similar effect in the drying of apricots, grapes, red pepper, and mulberry [52, 61, 62]. Pretreatment of dried fruit with antibrowning agent was found to enhance the moisture diffusivity, which is the movement of moisture through the grapes during the drying process [6]. Rehydration capacity helps to determine how well the dried grapes can absorb water and regain their original texture and flavor [63]. Table 5 shows that treated samples had an average rehydration percentage of 25.41 ± 2.38% which was higher than nontreated samples (9.98 ± 0.83%). For the effects of drying and pretreatment on the nutritional and functional quality of raisins, the results show that both TPC and TFC were significantly influenced by pretreatments (*p* < 0.05) compared to the nontreated samples. The level of TPC (135.79 ± 13.17 mg AGE/100 g DW) and TFC content (57.81 ± 3.08 mg Qeq/100 g DW) was higher by 34.50% and 33.62%, respectively, compared to the control samples. This difference can be explained by the fact that antibrowning agent used can limit the degradation of TPC by deactivation of PPO and POD enzymes responsible for degradation of phenolic compounds [64]. Browning rate parameters and HMF (indicator of the Maillard reaction) have been studied (Table 5). The control samples recorded high HMF values (77.60 ± 1.03 mg/100 g) and a browning rate (42.72%) compared to the samples treated with 5% of Na_2_S_2_O_5_. Thus, it can be concluded that browning is primarily caused by nonenzymatic browning (Maillard's reaction) at optimal conditions.

### 3.6. Microbial Quality Evaluation of Grapes under Optimal Conditions Stored for One Year

While research has extensively explored the dehydration mechanisms leading to the inactivation and resilience of microorganisms during drying [65], a growing concern is the presence of pathogens such as Salmonella in dehydrated food products. This concern is further supported by the work of Bourdoux et al. [66], which provides an overview of studies examining the presence of microorganisms like *S. Typhimurium*, *S. anatum*, and *coliforms*, on various fruits, vegetables, herbs, and spices resulting from different drying techniques. This is why it was crucial to evaluate the microbial quality of our raisins. Based on the results presented in Table 6, the microbial quality of dried grapes under optimum conditions stored for one year at room temperature was evaluated. The total viable count was 400 UFC/g (in dried grapes with 5% Na_2_S_2_O_5_) which was less than the bacterial growth observed in the control sample (1460 UFC/g). No Enterobacter, coliforms, or E. coli were detected in any of the samples, which is in accordance with the maximum tolerated value of 10^2^ UFC/g listed by [67]. Pathogenic bacteria such as Salmonella and Listeria were also not detected in grape samples (Table 6). The yeast and mold count never exceeded 100 UFC/g, which is consistent with international standards and the maximum tolerated value of 10^4^ UFC/g. These results were far more less than those published by [68], who study the microbiological quality of raisin (local and imported) consumed in Morocco; [68] testified that the counts of total coliforms (1.8 × 10^3^ to 1.5 × 10^4^ UFC/g) and fecal coliforms (1.1 × 10^3^ to 5 × 10^3^ UFC/g) were found to be high in most varieties of raisins, which could be attributed to poor conditions during preparation, transport, and marketing. The presence of yeasts in the samples of raisins was also found with high counts. For the identification by Maldi-Tof-MS, we managed to identify four species of Bacillus family with an accuracy more than 90%: *Bacillus pumilus*, *Bacillus subtilis*, *Bacillus amyloliquefaciens*, and *Cytobacillus horneckiae*. Other species were detected but with low precision (less than 70%); we did not mention them in this study.

## 4. Conclusion

Grape's conservation is challenging due to its short shelf life and susceptibility to spoilage. Drying is a common technique used in agrifood industry, but insufficient control in the drying process can lead to damaged grapes. This study demonstrated that using optimized condition (blanching for 5 minutes at 1% of NaOH solution, drying at 70°C with 30% of relative moisture) and an appropriate concentration of antibrowning agent (5% metabisulfite concentration) can lead to better preservation of appearance and quality of grapes vr. *Thompson Seedless*. These conditions resulted in a shorter drying time and lead to improved preservation of total phenol content, total flavonoid content, and color, while meeting international standards for SO_2_ content and microbiological safety.

## Figures and Tables

**Table 1:** Operating variables and ranges applied for drying optimization of grapes *Thompson Seedless grapes* (*Vitis vinifera L*.).

Variables	Symbol	Level	
		-1	+1
NaOH concentration (%)	*X*_1_	0.5	1
Na_2_S_2_O_5_ concentration (%)	*X*_2_	2	5
Temperature (°C)	*X*_3_	60	70
Relative humidity (%)	*X*_4_	30	40

NaOH: sodium hydroxide; Na_2_S_2_O_5_: sodium metabisulfite.

**Table 2:** Effect of NaOH concentration, antibrowning agent concentration, temperature, and relative humidity on physical chemical parameters of dried grapes according to a 2^4^ experimental design.

Order of running experiments	Level value of each variable in the experimental run				Responses			
	*X*_1_ (NaOH concentration (%))	*X*_2_ (Na_2_S_2_O_5_ concentration (%))	*X*_3_ (temperature (°C))	*X*_4_ (relative humidity (%))	*Y*_1_ (drying time (hours)	*Y*_2_ (color intensity change)	*Y*_3_ (chromaticity *C*^∗^)	*Y*_4_ (browning rate (%))
1	-1	-1	-1	-1	39	25.9	8.6	44.4
2	1	-1	-1	-1	38	25.7	8.9	44.4
3	-1	1	-1	-1	39	24.7	11.7	44
4	1	1	-1	-1	38	21.8	12.9	37.4
5	-1	-1	1	-1	24	23	9.8	35.9
6	1	-1	1	-1	23	22.5	10.9	35
7	-1	1	1	-1	24	19.7	14.8	32.6
8	1	1	1	-1	23	13.5	18.6	14.5
9	-1	-1	-1	1	43	24.3	9.2	39.7
10	1	-1	-1	1	42	23.6	9.8	38.7
11	-1	1	-1	1	43	21.4	12.8	34.3
12	1	1	-1	1	42	22.3	11.6	36.6
13	-1	-1	1	1	30	25.6	10.4	46
14	1	-1	1	1	29	25.9	10.1	46.4
15	-1	1	1	1	30	20.5	14	35
16	1	1	1	1	29	20.5	13.7	34.6

**Table 3:** Physicochemical and biochemical characterization of commercially mature *Thompson Seedless* grapes (*Vitis vinifera L*.) from different regions prior to drying.

	El Hajeb city	Meknes city	Nador city	Sig
Water content (%)	81.33 ± 1.20^a^	81.77 ± 0.67^a^	79.47 ± 1.69^a^	n.s
pH	4.09 ± 0.30^a^	4.35 ± 0.24^a^	4.24 ± 0.50^a^	n.s
Total soluble solids (°Brix)	16.37 ± 1.02^a^	25.33 ± 0.57^b^	19.03 ± 0.25^c^	^∗∗∗^
Chlorophyll a (mg/100 g FW)	268.34 ± 10.82^a^	324.72 ± 11.37^b^	446.96 ± 12.08^c^	^∗∗∗^
Chlorophyll b (mg/100 g FW)	677.51 ± 10.82^a^	292.45 ± 11.37^b^	1350.46 ± 12.08^c^	^∗∗∗^
Total chlorophyll (mg/100 g FW)	932.49 ± 10.82^a^	603.76 ± 11.37^b^	1782.13 ± 12.08^c^	^∗∗∗^
Total polyphenol content (mg GAE/100 g FW)	100.28 ± 3.70^a^	170.61 ± 5.60^b^	80.33 ± 4.60^c^	^∗∗∗^
Total flavonoid content (mg Qeq/100 g Fw)	20.55 ± 2.90^b^	35.30 ± 2.45^a^	17.43 ± 1.90^b^	^∗∗∗^
PPO activity (UIA/g.min)	17.14 ± 1.18^a^	18.95 ± 1.70^a^	14.59 ± 0.79^b^	^∗^
POD activity (UIA/g.min)	11.15 ± 1.68^a^	10.80 ± 1.70^a^	10.21 ± 0.26^a^	n.s
Chroma *C*^∗^	21.18 ± 0.52^a^	21.47 ± 0.15^a^	21.41 ± 0.34^a^	n.s
Yellow index	68.94 ± 3.74^a^	71.49 ± 1.10^a^	71.30 ± 1.61^a^	n.s

PPO: polyphenol oxidase; POD: phenol peroxidase; FW: fresh weight; GAE: gallic acid equivalent; Qeq: quercetin equivalent; signification levels: ^∗^*p* < 0.05, ^∗∗^*p* < 0.01, and ^∗∗∗^*p* < 0.001; n.s: nonsignificant (*p* > 0.05). Columns with the same letter belong to the same group.

**Table 4:** Estimated effects of the 2^4^-factorial design for the drying of grapes *Thompson Seedless grapes* (*Vitis vinifera L*).

Effect	Value of effect			
	*Y*_1_ (drying time (hours))	*Y*_2_ (color intensity change)	*Y*_3_ (chromaticity *C*^∗^	*Y*_4_ (browning rate (%))
*Average effect*	33.5 ± 0.0	22.55 ± 0.32^∗^	11.73 ± 0.23^∗^	37.46 ± 0.91^∗^
*Main effects*				
NaOH	−0.5 ± 0.0	−0.58 ± 0.32	0.32 ± 0.23	−1.51 ± 0.91
Na_2_S_2_O_5_	0 ± 0.0	−2.00 ± 0.32^∗^	2.02 ± 0.23^∗^	−3.84 ± 0.91^∗^
Temperature	−7 ± 0.0	−1.15 ± 0.32^∗^	1.05 ± 0.23^∗^	−2.46 ± 0.91^∗^
Relative humidity	2.5 ± 0.0	0.45 ± 0.32	−0.28 ± 0.23	1.44 ± 0.91
*Two-factor interactions*				
NaOH ^∗^ Na_2_S_2_O_5_	0 ± 0.0	−0.44 ± 0.32	0.11 ± 0.23	−1.33 ± 0.91
NaOH ^∗^ temperature	0 ± 0.0	−0.21 ± 0.32	0.21 ± 0.23	−0.85 ± 0.91
Na_2_S_2_O_5_^∗^ temperature	0 ± 0.0	−0.84 ± 0.32^∗^	0.46 ± 0.23	−1.98 ± 0.91
NaOH ^∗^ relative humidity	0 ± 0.0	0.643 ± 0.32	−0.47 ± 0.23	1.68 ± 0.91
Na_2_S_2_O_5_^∗^ relative humidity	0 ± 0.0	0.16 ± 0.32	−0.45 ± 0.23	0.05 ± 0.91
Temperature ^∗^ relative humidity	0.5 ± 0.0	1.26875^∗^ ± 0.32	−0.45 ± 0.23	4.05^∗^ ± 0.91

^∗^Signification levels (*p* < 0.05). NaOH: sodium hydroxide; Na_2_S_2_O_5_: sodium metabisulfite.

**Table 5:** Evaluation of quality attribute between treated and untreated grape samples dried under optimal conditions (5 min blanching in a solution of NaOH at 1% concentration; *T* = 70°C; RH = 30%).

Antibrowning agent		Physicochemical and biochemical characteristic								
Agent	Concentration	Water content (%)	*a*_w_	Rehydration rate	Browning rate	TPC (mg GAE/100 g DF)	TFC (mg Qeq/100 g DW)	HMF (mg/100 g DW)	SO_2_ content (mg of S0_2_/kg DF)	PPO residual activity (%)
Control	0%	23.55 ± 1.15^a^	0.69 ± 0.05^a^	9.98 ± 0.83	42.72 ± 0.00^a^	88.98 ± 1.22^a^	19.44 ± 0.76^a^	77.60 ± 1.03^a^	nd	2.70 ± 0.09^a^
Na_2_S_2_O_5_	5%	17.90 ± 1.80^b^	0.52 ± 0.06^b^	25.41 ± 2.38	14.48 ± 3.14^c^	135.79 ± 13.17^b^	57.81 ± 3.08^c^	12.40 ± 0.62^b^	580 ± 23.0^a^	0.58 ± 0.04^c^
Sig		^∗∗^	^∗∗^	^∗∗^	^∗∗∗^	^∗∗^	^∗∗∗^	^∗∗∗^	^∗∗∗^	^∗∗∗^

Na_2_S_2_O_5_: sodium metabisulfite; *E*^∗^: color intensity change; *C*^∗^: chromaticity; TPC: total phenol content; TFC: total flavonoid content; HMF: hydroxymethylfurfural; nd: not detected; SO_2_: sulfur dioxide content; PPO: polyphenol oxidase; signification levels: ^∗^*p* < 0.05, ^∗∗^*p* < 0.01, and ^∗∗∗^*p* < 0.001. Columns with the same letter belong to the same group.

**Table 6:** Evaluation of microbiological quality of dried grapes under optimum conditions (5 min blanching in a solution of NaOH at 1% concentration; *T* = 70°C; RH = 30%; 5% of Na_2_S_2_O_5_) after one year of storage at room temperature.

	CBT	Entero	Coliforms	E. coli	Lactic bacteria	Bacillus spp.	Yeasts	Molds	Sulfite reducing clostridia	Salmonella spp.	L. monocytogenes
control	1460	<10	<10	<10	<10	<10	<100	<100	<10	nd	nd
5% Na_2_S_2_O_5_	400	<10	<10	<10	<10	<10	<100	<100	<10	nd	nd

CBT: total bacterial load; Entero: Enterobacteriaceae; nd: not detected. Values of the loads are expressed as log UFC/g and UFM/g.

## Data Availability

The datasets generated during and/or analyzed during the current study are available from the corresponding author on reasonable request.
